# Two-point-based binary search trees for accelerating big data classification using KNN

**DOI:** 10.1371/journal.pone.0207772

**Published:** 2018-11-26

**Authors:** Ahmad B. A. Hassanat

**Affiliations:** IT Department, Mu'tah University, Mutah–Karak, Jordan; Nanjing University of Information Science and Technology, CHINA

## Abstract

Big data classification is very slow when using traditional machine learning classifiers, particularly when using a lazy and slow-by-nature classifier such as the k-nearest neighbors algorithm (KNN). This paper proposes a new approach which is based on sorting the feature vectors of training data in a binary search tree to accelerate big data classification using the KNN approach. This is done using two methods, both of which utilize two local points to sort the examples based on their similarity to these local points. The first method chooses the local points based on their similarity to the global extreme points, while the second method chooses the local points randomly. The results of various experiments conducted on different big datasets show reasonable accuracy rates compared to state-of-the-art methods and the KNN classifier itself. More importantly, they show the high classification speed of both methods. This strong trait can be used to further improve the accuracy of the proposed methods.

## Introduction

Over the last decade, the ever-increasing worldwide demand for communications and information of all kinds has resulted in dramatic developments in information technology which facilitates data sharing and communication universally [1]. These worldwide technological interactions among human beings create unprecedented large volumes of data, which lately is referred to as “big data” [2]. Big data renders technological hardware and software, to some extent, powerless to process such a huge amount of data effectively and promptly. For example, the common Microsoft Excel software cannot open a file that exceeds 2 GB, which is problematic when one takes into consideration that most big data files are normally much larger than 2GB.

Most of the distinguished machine-learning algorithms require an inordinate amount of time to be trained on such a big data files. For example, the K-nearest neighbors classifier (KNN) took more than 6 weeks to classify one dataset using high performance computing machines. Therefore, big data files require groundbreaking thinking to provide the means to overcome the data size problem and to facilitate the process of obtaining and analyzing vital information, which is important for new applications [3].

The KNN classifier is one of the most common and easy-to-implement classifiers in the machine learning domain, achieving competitive results compared with the most complex methods, and sometimes it is the only available choice, for example when used for content-based image retrieval (CBIR) [4] and [5]. However, it is a very slow classifier and a lazy learner. For testing any example, the KNN classifier cannot produce a small fixed-size training model to be used. Instead, it uses the complete training data each time. Given a training set of *n* examples, in *d* dimensional feature-space, the running cost to classify one example is O(*n*.*d*) time, and since we have the blessing or curse of big data, where *n* and/or *d* are relatively large values, the KNN becomes impractical, especially if it is used for an online application. The blessing of big data sets includes their ability to provide a rich source of information to the classifiers for a better learning, while the curse of big data sets includes their very large sizes.

Fortunately, the researchers in big data classification domain have made numerous efforts to find practical solutions for such a problem. Remarkable efforts have been made to enhance the speed and efficiency of the KNN classifier to be applied to big data. We will shed light on the most recent work, which deals with big data classification using KNN, such as the work of Maillo et al.[6], who proposed a parallel map-reduce implementation based on mapping the training set examples and followed this by reducing the number of examples that are interconnected to a test sample (MR-kNN). The reported results were similar to that of the KNN's but much faster–about 16 to 149 times when tested on one dataset of 1 million examples. The speed of the proposed algorithm depends mainly on the number of maps (16–256) and K neighbors used. The mapping phase consumes a substantial amount of time, mainly with large values of K. This inspiring work has been developed further by the same authors [7], where they propose a new KNN based on Spark (kNN-IS) which is alike to the map-reduce technique but with using multiple reducers to speed up the process. The size of dataset used was up to 11 million examples. Their Spark package can be downloaded at http://spark-packages.org/package/JMailloH/kNN_IS.

Based on clustering the training set using K-means clustering algorithm, Deng et al. [8] proposed two methods to increase the speed of KNN. The first used random clustering (RC-KNN) and the second used landmark spectral clustering (LC-KNN). When obtaining the related cluster, both apply the sequential KNN to test the input example with a smaller set of examples. Both algorithms were assessed on nine big datasets showing reasonable approximation, taking into consideration that the accuracy of the KNN depends on the number of clusters used.

Recently, an additional clustering approach was developed by Gallego et al. [9], who proposed two clustering methods to accelerate the speed of the KNN called cKNN and cKNN+. They are similar; however, the latter uses a cluster augmentation process. The reported average accuracy of all the big datasets employed was in the range of 83 to 90%, dependent on the number of clusters used; however, these results improved considerably when they used Deep Neural Networks for learning a suitable representation for the classification task.

In the same year, Wang et al. [10]proposed two multivariate decision tree classifiers to solve the problem (of the slow speed of big data classification using KNN). The first uses a random partition (MDT1), and the second uses Principal Component Analysis (PCA)-partitioned method (MDT2). Both are used separately to partition the training data used. These classifiers enable the training time to be short and the classification accuracy to be high for large-scale datasets. Full binary-balanced trees are generated, based on two simple means of creating multivariate combination weights and employing a median-based method to select the divide value.

Most of the proposed work in this domain depends mainly on a ‘divide and conquer’ algorithmic principle–this is a reasonable and rational approach to be used with big datasets. Therefore, most of these approaches are based on clustering, splitting, or partitioning the data to reduce its enormous size to a manageable size that can be targeted later by the KNN. However, such approaches inherit a key problem that is the determination of the best number of clusters, as more clusters means less data, and therefore faster testing, but less data also means less accuracy, as the remaining examples might not be related to the tested example. On the other hand, a small number of clusters specifies a large number of examples for each cluster, which increases the accuracy but slows down the classification process. We can call this phenomenon the ‘clusters-accuracy-time dilemma’. Another related problem is finding the best k for the KNN, which is beyond the scope of this paper. For more on this see [11] and [12]. In this paper we will work on k = 1 i.e. the nearest neighbor (1NN).

Regardless of the high quality and quantity of the proposed methods in this domain, there is still room for improvement in terms of accuracy and time consumed for both training and testing. In this paper, we avoid the clustering approach to speed up big data classification using KNN, because of the clusters-accuracy-time dilemma. As an alternative, we propose a new approach based on storing the training examples into a binary search tree, to be used later by the KNN classifier. In doing so, we are attempting to achieve the goal of this paper, which is related to accelerating big data classification using the KNN classifier. Therefore, searching the tree for an example should be faster than searching the whole training data, and faster than searching the examples within a specific cluster, particularly when a cluster has many examples.

In this paper we propose two methods to create the binary search tree. Both of the created trees depend largely on finding two local examples (points), which are used to sort the other examples based on their similarity to these local points. The first tree-based method chooses the local points randomly, and the second chooses them based on their similarity to global points. Throughout this paper, we will use the words ‘point’ and ‘example’ interchangeably to refer to the same thing, which is the feature vector in d-dimensional feature space obtained from training or testing datasets.

The rest of this paper will introduce the proposed methods and the dataset used, as well as the results obtained from the comparison of the proposed methods with other state-of-the-art methods, namely MR-kNN, kNN-IS, RC-KNN, LC-kNN, cKNN, cKNN+, MDT1, and MDT2.

## Two-point-based binary search trees

Finding the nearest neighbor(s) example in machine learning datasets is time-consuming; typically, this running time is not acceptable, particularly when using big machine learning datasets. In this paper, we propose the use of a binary search tree (BST) to create some kind of indexing for the examples (points) of machine learning datasets. This BST sorts the points based on their similarity to two local points (P1 and P2). These points are two examples from the train dataset itself, and they are changed at each level and node of the BST. Here we propose two methods to choose these points at each level/node. The first chooses them randomly, and we call it Random-Points-based binary search tree (RPBST). The second method chooses P1 based on its similarity to the global extreme minimum point, and P2 based on its similarity to the global extreme maximum point in the training dataset–we call this method Extreme-Points-based binary search tree (EPBST).

The training phase of both algorithms ends by creating their BSTs, which makes searching for a test example much faster than the simple sequential search used by naïve KNN—we call it naïve here because of the unacceptable time consumed by this algorithm in big datasets. Both methods create their BST by storing the examples depending on their similarities to P1 and P2. Those which are similar to P1 are stored with the left child of a node in a lower level, and those which are similar to P2 are stored with the right child of the same node in the same lower level. The BST is then built to the leaves in the same way, recursively. It is expected to have only one example stored with each leaf node. However, in some cases we have more than one example, particularly if we have duplicate examples, and/or when two or more examples exhibit the same similarity to P1 or P2. Both methods employ Euclidian distance for measuring similarity because the KNN algorithm that we are approximating uses this distance metric in general, seeing as most of the state-of-the-art methods in this domain use this distance metric.

The test phases of the both methods use the same approach of searching the created BST—for a test example—to the leaf, and then they use the naïve KNN classifier to classify the test example based on those examples stored in the found leaf node. If there is only one example in the found leaf, there is no need to apply KNN or 1NN, for obvious reasons, but in our experiments we did so anyway, because sometimes more than one example are found in a leaf-node.

### EPBST

Given a training dataset with *n* feature e vectors (FV) and *d* features, RPBST builds its BST by finding the two extreme (minimum and maximum) global points GP1 and GP2 as follows:

$${GP1}_{i}=\min_{j=1\;\mathrm{to}\;n}\left({FV}_{ji}\right)$$ (1)

and

$${GP2}_{i}=\max_{j=1\;\mathrm{to}\;n}\left({FV}_{ji}\right)$$ (2)

where *i* is the index of the feature for each FV, and j is the index of each FV in the training dataset.

Therefore, GP1 and GP2 are virtual points in the d-dimensional feature space as follows:

$$GP1=({GP1}_{1},{GP1}_{2},{GP1}_{3},\ldots ,{GP1}_{d})\;\mathrm{and}\;GP2=({GP2}_{1},{GP2}_{2},{GP2}_{3},\ldots ,{GP2}_{d})$$

After calculating the global extreme points, we need to calculate the local extreme points (P1 and P2) for each node as follows:

$${P1=\mathrm{argmin}}_{j=1^{st}index\;to\;m}\left(ED({FV}_{j},GP1)\right)$$ (3)

and

$${P2=\mathrm{argmin}}_{{j=1}^{st}index\;to\;m}\left(ED({FV}_{j},GP2)\right)$$ (4)

where *ED* is the Euclidean distance between each *FV* in a node and both of *GP1* and *GP2*, *j* is the index of each FV in the training dataset and *m* is the index of the last FV stored in a node.

After calculating P1 and P2 for the root node, the training phase algorithm of EPBST stores the indexes of all FVs from the training dataset in the root of its BST, and it calculates the Euclidean distance between all FVs and both of P1 and P2 to sort the FVs into minimum and maximum FVs based on their similarity to P1 (min) and P2 (max). The indexes of the minimum FVs are stored with the left child node, and the others with the right child node. EPBST continues to calculate local extreme points and categorizes the FVs recursively until it gets only one FV in a leaf-node or a number of FVs with the same similarity to either P1 or P2 in the upper level. Algorithm 1 describes building the BST for EPBST (the training phase).

The time complexity of building the BST is O(4*dn* log *n+ 2dn*), where (2*d*) is the time consumed to find P1 and P2, and another (2*d*) is time consumed by comparing the FVs to P1 and P2 at each node, and the (*2d*.n) is time consumed by the process that finds the global extreme points. However, if *n* >> *d*, the training time complexity can be approximated to *O(n* log *n*+*n*). Obviously the *d* extra running cost comes from calculating the Euclidian distance between the FVs and P1 and P2. With some optimization of the code, these distances can be calculated once and used along building the BST. By then the time complexity will be reduced to O (*2dn* log *n*+ *2d n*) regardless of the size of d.

- - - - - - - - - - - - - - - - - - - - - - - - - - - - - - - - - - - - - - - - - - - - - - - - - - - - - - - - - - - - - - - - -

Algorithm 1: Training Phase (building) of EPBST.

Input: Numerical training dataset DATA with *n* FVs and *d* features.

Output: A Root pointer to the EPBST.

- - - - - - - - - - - - - - - - - - - - - - - - - - - - - - - - - - - - - - - - - - - - - - - - - - - - - - - - - - - - - - - - -

GP1 ←**min**(FVs) //Eq 1

GP2 ←**max**(FVs) //Eq 2

Create a BST Node → RootN

RootN.Examples←FVs //store indexes of FV from training dataset

RootN.P1← argmin(ED (RootN.Examples, GP1)) // Eq 3

RootN.P2←argmin(ED (RootN.Examples, GP1)) //Eq 4

RootN.Left = Null

RootN.Right = Null

**Procedure** BuildBST(Node←RootN)

        **for *each*** *FV*_*i*_ ***in*** *Node*, **do**

                D1←ED(*FV*_*i*_, Node.P1)

                D2←ED(*FV*_*i*_, Node.P2)

                **If** (D1<D2)

                        Add index of *FV*_*i*_ *to* Node.Left.Examples

                **else**

                        Add index of *FV*_*i*_ *to* Node.Right.Examples

        **end**

        **if** (Node.Left = = Null **or** Node.Right = = Null)

                **return** //this means a leaf node

        **end**

        BuildBST (Node.Left) //recursive call

        BuildBST (Node.Right) //recursive call

**end Procedure**

**return** RootN

- - - - - - - - - - - - - - - - - - - - - - - - - - - - - - - - - - - - - - - - - - - - - - - - - - - - - - - - - - - - - - - - -

When calculating P1 and P2, we sometimes get the same example, which is most similar to both GP1 and GP2, in our code; we enforced such an example to form P1, and the next minimum to form P2.

To test an example *E* in the test phase, starting from the root node of the created BST as a current node, EPBST calculates the ED between E and both of P1 and P2. If E is more similar to P1 than P2, the current node becomes the left-child node, otherwise it becomes the right-child node, and recursively searches the BST to the leaf to find one or more FVs. These will be used by the naïve KNN algorithm to predict the class of the inputted test example. Searching the tree costs O(2*d*.log *n*) time for each test example because the average depth of the BST created in the training phase is about (log *n*), as we split the training examples by enforcing at least one example per each node, similar to the well known binary search tree. The (2*d*) is time consumed by the Euclidian distance as it uses all the features (d) to get the distance between E and both of P1 and P2.

In addition to the search time, the time complexity of our implementation of the KNN alone becomes O(d) for each tested FV if there was only one FV in the leaf node found, which is very fast, and even this time can be neglected as we may not use the KNN at all by just announcing the class of the example found in the leaf node reached. However, sometimes the leaf nodes contain more than one example. This is due to duplicate examples, however, they are of small numbers and more likely they belong to the same example, so the use of KNN is not too important, leaving the search time dominating the complexity of the proposed method in the test phase. Algorithm (2) shows the test phase of NBT.

- - - - - - - - - - - - - - - - - - - - - - - - - - - - - - - - - - - - - - - - - - - - - - - - - - - - - - - - - - - - - - - - -

Algorithm 2: Testing Phase of EPBST.

Input: Numerical testing dataset TESTDATA with *n* FVs and *d* features.

Output: Test Accuracy.

- - - - - - - - - - - - - - - - - - - - - - - - - - - - - - - - - - - - - - - - - - - - - - - - - - - - - - - - - - - - - - - - -

Accuracy←0

**for *each*** *FV*_*i*_ ***in*** *TESTDATA*, **do**

        **Procedure** GetNode(Node←RootN, *FV*_*i*_)

                D1←ED(*FV*_*i*_, Node.P1)

                D2←ED(*FV*_*i*_, Node.P2)

                **if** (D1<D2 **and** Node.Left)

                        **return** GetNode(Node.Left, *FV*_*i*_)

                **else if (**D2 ≤ D1 **and** Node.Right**)**

                        **return** GetNode(Node.Right,*FV*_*i*_)

                **else**

                        **return** Node

                **end**

        **end Procedure**

        **Procedure** KNN(FV_i_, Node)

                Array Distances;

                **for *each*** *fv*_*j*_ ***in*** *Node*, **do**

                        Distances[j] ←ED[*fv*_*j*_, FV_i_]

                **end**

                index ←argmin(Distances[j])

                c ←Class(*fv*_index_)

                ***return*** *c*

        **end Procedure**

        **if** c = = Class(FV_i_)

                Accuracy←Accuracy+1

        **end**

**end**

Accuracy←Accuracy/n

**return** Accuracy

- - - - - - - - - - - - - - - - - - - - - - - - - - - - - - - - - - - - - - - - - - - - - - - - - - - - - - - - - - - - - - - - -

### RPBST

Similarly to EPBST, this method trains a BST where each node stores the indexes of a number of training examples based on their similarity to two local points (P1 and P2), which are stored in a parent node. RPBST chooses these points randomly from the training data. To build the BST, the RPBST employs the Euclidian distance to find similar FVs to the P1 to store them in the left-child node, and those similar to P2 are stored in the right-child node of a parent node having P1 and P2. The RPBST proceeds to sort all the FVs of a training data and store them in a BST recursively, until no further sorting is possible, i.e. one example per node, which becomes a leaf node. However, sometimes a training dataset will have similar or duplicate examples—this is common in big data, but if there are any, they are few.

Similar to EPBST building the BST by the RPBST takes O(*2d n* log n) training time, the (2*d*) is time consumed by the ED between FVs and both of P1 and P2. In practice, however, it takes less time (about half) compared to the EPBST, because it chooses P1 and P2 randomly without the extra cost (2d) of comparing all FVs to the global extreme points (GP1 and GP2), and without the other extra time (2dn) which is needed to find both GP1 and GP2.

The testing phase is exactly the same as that of the EPBST: given the trained BST and a test Example *E*, the RPBST searches its BST for *E* starting from the root node, comparing its distance to the local points (P1 and P2), which are stored in each node, and finds its path left/right recursively, until it finds a leaf node. The FVs found in a leaf node are used along with E by the naïve KNN to predict the class of E, although this is not needed as we have only one example per leaf node or a very small number compared to the size of the training dataset n. Algorithm 2 can be used for this purpose by replacing the BST of the EPBST with that of the RPBST. Consequently, the time complexity of the test phase of the RPBST is the same as that of the EPBST. Searching the BST created by RPBST costs O(2*d* log *n*) in all cases, which is very fast with a small number (*d*) of features.

Building the tree of the RPBST can be described by the same Algorithm 1 if we removed steps 1 and 2 (which calculate the global extreme points), and replaced steps 5 and 6 by choosing P1 and P2 randomly, and not based on their similarity to the global extreme points. Space needed to build the BST for each method is O(n log n), as we store all the indexes of the training data in each level in the tree. We do this because we want the resultant BST to be for general use, and not only for the purposes of this study, as we are thinking of using the upper levels to enhance the accuracy and to find the K neighbors there. However, this paper concerns only the 1NN classifier.

### Data

To evaluate and compare the proposed methods we used some of the well-known machine learning datasets, which were obtained from either Machine Learning Repository [13] or LIBSVM Data [14]. These datasets are normally used for evaluating machine learning methods, and therefore we reused them for comparison purposes. We used intermediate and large numerical datasets, with different dimensions and sizes. All datasets used contain numerical data, integers and/or real numbers. Table 1 shows the description of these dataset.

**Table 1:** Description of datasets used for evaluation and comparison.

Dataset	Size	Dimensions	Type	#Class
HIGGS	11000000	28	Real	2
SUSY	5000000	18	Real	2
Poker	1025010	11	Integers	10
Covtype	581012	54	Integers	7
Mnist	70000	784	Integers	10
Connect4	67557	42	Integers	3
Nist	44951	1024	Integers	26
Letter	20000	16	Real	26
Homus	15199	1600	Integers	32
Gisette	13500	5000	Integers	2
Pendigits	10992	16	Integers	10
Usps	9298	256	Real	10
Satimage	6435	36	Real	6

## Results and discussions

We conducted several experiments to evaluate both EPBST and RPBST after programming these proposed methods using the MS VC++.Net framework to classify the datasets described in the data section. We utilized Azure's cloud platform for high-performance computing (https://portal.azure.com). The virtual machine used was built on 16 Intel CPUs @ 2.3 GHz with 32GB RAM. Table 2 shows some characteristics of the BST built by either EPBST or RPBST.

**Table 2:** The resultant BSTs after applying EPBST and RPBST in the training phase on 80% of data, time in (ms).

database	Characteristic	RPBST	EPBST
Poker	Building Time	23212	45187
	#Examples	820008	820008
	#Leaves	817214	817223
	Max#Ex in a leaf	95	63
	maxDepth	72	47
	Avg#Ex in a leaf	1.003	1.003
	Log#Ex	19.6	19.6
SUSY	Building Time	427656	844253
	# Examples	4000000	4000000
	#Leaves	4000000	4000000
	Max#Ex in a leaf	1	1
	maxDepth	81	87
	Avg#Ex in a leaf	1	1
	Log#Ex	21.9	21.9
HIGGS	Building Time	1003972	2278469
	# Examples	8800000	8800000
	#Leaves	8452172	8446460
	Max#Ex in a leaf	777	2086
	maxDepth	184	121
	Avg#Ex in a leaf	1.0412	1.0419
	Log#Ex	23.0691	23.0691

#Examples represents the number of example from the training dataset used (n), #Leaves represents the number of leaves in the resultant BST, "Max#Ex in a leaf" is the maximum number found in a leaf node, maxDepth is the maximum depth of the tree, "Avg#Ex in a leaf" is the average of number of examples per leaf node and Log#Ex is log n.

The most interesting thing to note from Table 2 is the maximum depth of both of the resultant BSTs, which is not much greater than log *n*, as the average depth is almost equal to log *n* of each dataset, which will speed up the testing phase using the naïve KNN classifier. Moreover, the training time of the EPBST is almost double that of the RPBST, as expected, since the EPBST uses ED to find P1 AND P2 at each node when compared to the GP1 and GP2 respectively, in addition to calculating the GP1 and GP2. Another hastening factor of both BSTs is the average number of examples in all leaf nodes, which is around 1 for all datasets used. Therefore the time complexity of the naïve KNN becomes O(d), separate from the search time, as stated in the previous section.

Our experiments’ settings were different based on the compared work, following those settings of the state-of-the-art methods to which they were compared. This is essential to be able to make valid comparisons with other methods, given that different researchers used various evaluation schemes on different datasets.

It is worth mentioning that the proposed algorithms had not benefited from the Multi CPUs available in the cloud platform used, because they were single-threaded algorithms; however, this high-performance computing platform allows for multiple experiments to run at the same time, which significantly reduced the time needed to conduct our experiments.

Since each of the previous methods used different hardware with differing computational power, we opted for measuring the speed-up factor of each algorithm similarly to [6] and [15], which is calculated by comparing the time consumed by an algorithm with that of the naive KNN algorithm at the same machine, using the same dataset and same number of examples as follows

$$Speedup(A,B)=\frac{Time(KNN,B)}{Time(A,B)}$$ (5)

where *A* is the algorithm whose speedup factor we want to calculate, *B* is the dataset used and the *Time* function returns the time consumed by an algorithm when tested on a specific dataset.

Both the MR-kNN [6]and kNN-IS [7] were evaluated on Poker and Susy datasets, using 5-fold cross-validation (CV). To compare our results with these methods we used the same CV on the same datasets. The accuracy results and speed-ups of these methods as well as the proposed ones are shown in Tables 3 and 4 respectively.

**Table 3:** Accuracy results of MR-KNN and KNN-IS compared to the proposed methods in the test stage with 5-fold-CV.

Method	Dataset	Accuracy	Avg RunTime(ms)
MR-KNN	Poker	0.502	804456
	SUSY	0.694	12367966
KNN-IS	Poker	0.502	102938
	SUSY	0.694	1900039
KNN	Poker	0.502	105475006
	SUSY	0.694	3258848811
Our RPBST	Poker	**0.534**	**43417**
	SUSY	**0.709**	**189763**
Our EPBST	Poker	0.519	101980
	SUSY	**0.709**	252378
Our KNN*	Poker	0.594	76414385
	Susy	?	2360966234

*Our KNN's time on Susy is approximated based on the ratio between the reported time consumed by KNN on the Susy and Poker datasets. As KNN took 30.9 times the time consumed on Poker, we multiplied this ratio with the time consumed by our KNN on poker, and the accuracy is still unknown as the algorithm took more than 6 weeks and we had to stop it. We mean by our KNN is the well-know naive KNN, which is implemented by us on the same machine that is used for evaluating the proposed methods. And the KNN is the one applied by the authors of the compared work—technically they are the same, but since they are applied on different machines, we had to distinguish the reported results of the KNN from our calculated results of the same KNN as the speeding factors of our proposed methods are dependent on our implementation of the KNN, and the speeding factors of the compared works are dependent on their implementation of the KNN. See Eq 5.

**Table 4:** Speed-up comparison of MR-KNN and KNN-IS, with 5-fold-CV.

Method	Poker	Susy
MR-KNN	131	263
KNN-IS	1025	1715
RPBST	**1760**	**12442**
EPBST	749	9355

The accuracy results recorded in Table 3 are the same for MR-KNN, KNN-IS and KNN because these algorithms are parallel-exact algorithms. Despite being approximate, the proposed methods outperform MR-KNN, KNN-IS and the naïve KNN in both accuracy and speed on both datasets used. A closer look at the data in Table 4 reveals an interesting note about the very high speed of the proposed methods, particularly, the RPBST, whose speed is about to approach twice the speed of the KNN-IS on the Poker datasets, more than seven times the speed of the same algorithm when tested on the SUSY dataset, and much higher than that of the MR-KNN on both datasets, provided that our proposed methods are single-threaded algorithms executed on a single CPU. Both the MR-KNN and KNN-IS were designed as parallel algorithms, and executed on a parallel computer with multi CPUs. The reason behind this significantly high speed is the use of the BST, whose average depth is almost the same as the logarithm of the size of the training data, and therefore speeds up the testing phase significantly.

We also compared the proposed methods with the RC-KNN and LC-KNN [8]These methods were evaluated using 10-fold-CV on a number of big machine-learning datasets. We followed the same experiment's setting on the same datasets to conduct a valid comparison. The accuracy results of the comparison and the speed-ups are shown in Tables 5 and 6 respectively.

**Table 5:** Accuracy results of RC-KNN and LC-KNN compared to the proposed methods in the test stage, with 10-fold-CV.

Dataset	RC-kNN	LC-kNN	kNN	RPBST	EPBST	our KNN
Usps	0.903	**0.936**	0.95	0.860	0.849	0.97
Mnist	0.722	0.839	0.86	0.850	**0.852**	0.97
Gisette	0.931	**0.953**	0.97	0.873	0.917	0.96
Letter	0.789	**0.950**	0.95	0.802	0.757	0.96
Pendigits	0.945	**0.972**	0.98	0.964	0.966	0.99
Satimage	0.860	**0.888**	0.91	0.868	0.860	0.90
Average	0.858	**0.923**	0.936	0.870	0.867	0.961

**Table 6:** Speed-up comparison of RC-KNN and LC-KNN, with 10-fold-CV.

Dataset	RC-KNN	LC-KNN	RPBST	EPBST
Usps	9.2	8.7	**218**	113
Mnist	8.2	6.8	**1161**	354
Gisette	9.3	7.6	**133**	21
Letter	6.1	6.0	**101**	41
Pendigits	3.1	3.0	**65**	16
Satimage	2.8	2.7	**56**	20

As can be seen from Tables 5 and 6, the accuracy results achieved by the proposed RPBST and EPBST are almost the same in average, competitive to those of LC-KNN and outperforming the RC-KNN in most datasets tested. As expected, the speedup factors obtained by the proposed methods are much higher than those of RC-KNN and LC-KNN, due to the power of the BSTs used in the test phase.

In addition, we compared our methods with the cKNN and cKNN+ [9], experimenting on the same datasets with the same 5-fold-CV used by both of cKNN and cKNN+. Table 7 shows the results of the proposed methods. Since there was no reported consumed time for both of the cKNN and cKNN+, we could not compare the speed of our methods with those of the cKNN and cKNN+. Moreover, the authors reported the average accuracy on all datasets used and not per dataset, which makes the comparison inadequate. However, we may compare with the reported average accuracies, which were almost the same for both of the cKNN and cKNN+, and were in the range of 83 to 90% depending on the number neighbors and clusters used. It is worth mentioning that [9]reported other excellent results reach 99% on average, but these significant results were achieved after applying Deep Neural Networks (DNN) for feature extraction on all the datasets used. Therefore, we did not compare our results with these as the data after feature extractions becomes different and biases the comparison.

**Table 7:** Classification accuracy comparison of RPBST, EPBST and KNN, with 5-fold-CV.

	Accuracy			Speed-up	
Database	RPBST	EPBST	KNN	RPBST	EPBST
Gisette	0.870	**0.913**	0.960	**33**	19
Letter	**0.800**	0.754	0.957	**72**	71
Homus	0.439	**0.560**	0.647	**134**	7
Satimage	**0.866**	0.860	0.904	**49**	41
Pendigits	**0.962**	0.961	0.994	**39**	29
Mnist	0.846	**0.848**	0.972	**991**	317
Usps	**0.856**	0.846	0.973	**159**	136
Nist	0.502	**0.539**	0.797	**660**	20
Average	0.768	**0.785**	0.901	**267**	80

As can be seen from Table 7, the proposed methods achieved reasonable results compared to those of the naïve KNN, and–on some datasets—the proposed methods outperformed the maximum average accuracy of both cKNN and cKNN+. Obviously, our proposed methods trade off some accuracy for extreme high speed. However, the speeding-up factor was not greatly significant as would be expected for some of these datasets. This is due to the large value of d, which is relatively higher than other datasets such as Homus and Gisette, whose dimensions are 1600 and 5000 respectively.

We finally compared the performances of the proposed methods with those of the MDT1, and MDT2, which were evaluated on 24 big datasets. However, we tested our methods on only 10 of these because we could not find some of the other datasets available anywhere, or we found some of them contained non-numerical data. The evaluation scheme of both of the MDT1 and MDT2 is based on hold-out-set, i.e. each dataset is split into two—one for testing and the other for training with a specific ratio. Wang et al. [10]evaluated these methods with different test/train ratios, which were in the range of 14 to 98% depending on the dataset used. Moreover, they pre-processed the datasets by removing redundant examples and reduced the dimensionality of the datasets using PCA. We could not reach these pre-processed datasets and therefore we experimented on the available original datasets, with the same test/train ratios used by Wang et al. Table 8 shows the comparison.

**Table 8:** Accuracy results of MDT1and MDT2 compared to the proposed methods in the test stage, with different test ratios.

Dataset	Test Ratio%	MDT1	MDT2	KNN	RPBST	EPBST	Our KNN
Poker	98	0.5073	**0.543**	0.5107	0.4869	0.4754	0.5106
SUSY	20	0.7289	**0.7491**	—	0.7097	0.7091	—
Covtype	20	0.8576	0.9010	—	**0.9291**	0.9168	0.9645
Letter	26	0.6160	**0.8052**	0.9568	0.7854	0.7448	0.9539
Pendigits	32	0.8509	0.9371	0.9774	0.9619	**0.9637**	0.9937
Satimage	31	0.7882	0.8555	0.8950	**0.8660**	0.8449	0.8935
Connect-4	20	0.6596	**0.6760**	0.6567	0.6398	0.6343	0.6109
Usps	22	0.6605	**0.8585**	0.9507	0.8411	0.8515	0.9766
Gisette	14	0.6170	0.9010	0.9580	0.8619	**0.9219**	0.951
HIGGS	20	0.5808	0.6002	—	0.5881	0.5854	—
Avg.	30.3	0.6867	0.7827	0.8436	0.7670	0.7648	0.8568

As can be seen from Table 8, the proposed methods have again performed almost the same in terms of accuracy, outperforming the MDT1 on most datasets, and the MDT2 on some datasets. The average accuracy rate of both of the proposed methods is significantly higher than that of the MDT1, and slightly less than that of the MDT2, provided that both the MDT1 and MDT2 were applied on pre-processed datasets with lower dimensionality. Such a pre-process does not only enhance the accuracy rate, but also it increases the speed of the algorithms, as can be seen form Table 9, where the MDT1 or MDT2 could beat the proposed methods in speed when tested on 3 datasets.

**Table 9:** Speed-up comparison of MDT1, and MDT2, with different test ratios.

Dataset	MDT1	MDT2	RPBST	EPBST
Poker	835	**927**	68	75
SUSY	-	-	-	-
Covtype	-	-	447	**699**
Letter	14	75	**390**	11
Pendigits	27	3	**36**	6
Satimage	13	7	**46**	11
Connect-4	**10745**	236	263	296
Usps	50	26	**171**	75
Gisette	**183**	16	147	20
HIGGS	-	-	-	-

Actually, this is the first time in our experiments that we have found an algorithm that is faster than both of the proposed methods. This is due to the fact that a) the datasets used by MDT1 and MDT2 are pre-processed and their dimensionalities were reduced, and this allows for faster ED calculations, and b) both methods used a binary tree (similar to ours) which split the data to a certain level, and this of course reduces the test time dramatically.

In summary, the previous presentation and discussion of the proposed RPBST and EPBST results show that these methods are very fast compared to other methods when classifying big data using the KNN approach, with reasonable accuracy rates when compared to other methods and the Naïve KNN classifier. This allows for building on these methods to further enhance the accuracy rates, trading off speed for accuracy. We also noted that both of the proposed methods perform almost equally in terms of accuracy, with some ups and downs for each. We justify this frequent behavior by the nature of the data itself: if the data is distributed in a way that allows the extreme points to correctly sort the examples in the BST, then the EPBST performs better, while conversely, the RPBST performs better. This natural behavior of the data sometimes reflects itself in the size, depth and shape of the resultant BST, which makes the speed as well as the accuracy of both of the proposed methods different from one dataset to another, as we found the RPBST is faster in most datasets, but also we found the EPBST is faster when tested on some other datasets. However, the training time for the RPBST was always much shorter (about half) than that of the EPBST, as justified above.

## Conclusions

In this paper we propose a new approach, which is based on sorting the feature vectors of the training data in a binary search tree, to speed up big data classification using the KNN approach. In order to do that we proposed two methods, both of which utilize two local points to store the examples based on their similarity to these local points, which are calculated for each node in the BST. The first method (EPBST) chooses the local points based on their similarity to the global extreme points, while the second method (RPBST) chooses the local points randomly.

The results of different experiments on various intermediate and big datasets, show reasonable accuracy rates, when compared to state-of-the-art methods and the naïve KNN classifier, and more importantly, they show the high classification speed of both methods. This strong trait can be used to hybridize and further enhance the proposed methods to achieve faster and more accurate algorithms.

It is also possible to increase the accuracy of the proposed methods by taking more examples from the higher levels of the tree, and not relying only on those found in a leaf node. This serves two purposes: first, it will increase the number of examples that can be used by the KNN classifier, thereby increasing the accuracy rate, and second, it allows for more (K) neighbors to be used, as one of limitations of this study, in addition to the fluctuate accuracy rates, is the use of 1NN with all of our experiments. This, of course, will increase the running cost but we do not have to worry much about it, since both of the proposed algorithms are significantly fast compared to other methods. These improvements will be addressed in our future work, along with other enhancements such as the investigation of using other distance metrics like Manhattan and Hassanat distances [16] and [17]. It is also possible to increase the accuracy of classification using different methods to store data points in the BST, such as the use of furthest-pair of points [18]. Such a method might increase the accuracy but this will be at the expense of the classification speed as shown in [19].
